# Lysyl oxidase-like 4 exerts an atypical role in breast cancer progression that is dependent on the enzymatic activity that targets the cell-surface annexin A2

**DOI:** 10.3389/fonc.2023.1142907

**Published:** 2023-04-04

**Authors:** Ni Luh Gede Yoni Komalasari, Nahoko Tomonobu, Rie Kinoshita, Youyi Chen, Yoshihiko Sakaguchi, Yuma Gohara, Fan Jiang, Ken-ich Yamamoto, Hitoshi Murata, I Made Winarsa Ruma, I Wayan Sumardika, Jin Zhou, Akira Yamauchi, Futoshi Kuribayashi, Yusuke Inoue, Shinichi Toyooka, Masakiyo Sakaguchi

**Affiliations:** ^1^ Department of Cell Biology, Okayama University Graduate School of Medicine, Dentistry and Pharmaceutical Sciences, Okayama, Okayama, Japan; ^2^ Faculty of Medicine, Udayana University, Denpasar, Bali, Indonesia; ^3^ Department of General Surgery & Bio-Bank of General Surgery, The Fourth Affiliated Hospital of Harbin Medical University, Harbin, Heilongjiang, China; ^4^ Department of Microbiology, Kitasato University School of Medicine, Sagamihara, Kanagawa, Japan; ^5^ Medical Oncology Department of Gastrointestinal Tumors, Liaoning Cancer Hospital & Institute, Cancer Hospital of Dalian University of Technology, Shenyang, Liaoning, China; ^6^ Department of Biochemistry, Kawasaki Medical School, Kurashiki, Okayama, Japan; ^7^ Faculty of Science and Technology, Division of Molecular Science, Gunma University, Kiryu, Gunma, Japan; ^8^ Department of General Thoracic Surgery and Breast and Endocrinological Surgery, Okayama University Graduate School of Medicine, Dentistry and Pharmaceutical Sciences, Okayama, Okayama, Japan

**Keywords:** breast cancer, lysyl oxidase, annexin A2, integrin, cancer microenvironment

## Abstract

**Background:**

LOX family members are reported to play pivotal roles in cancer. Unlike their enzymatic activities in collagen cross-linking, their precise cancer functions are unclear. We revealed that LOXL4 is highly upregulated in breast cancer cells, and we thus sought to define an unidentified role of LOXL4 in breast cancer.

**Methods:**

We established the MDA-MB-231 sublines MDA-MB-231-LOXL4 mutCA and -LOXL4 KO, which stably overexpress mutant LOXL4 that loses its catalytic activity and genetically ablates the intrinsic *LOXL4* gene, respectively. *In vitro* and *in vivo* evaluations of these cells’ activities of cancer outgrowth were conducted by cell-based assays in cultures and an orthotopic xenograft model, respectively. The new target (s) of LOXL4 were explored by the MS/MS analytic approach.

**Results:**

Our *in vitro* results revealed that both the overexpression of mutCA and the KO of LOXL4 in cells resulted in a marked reduction of cell growth and invasion. Interestingly, the lowered cellular activities observed in the engineered cells were also reflected in the mouse model. We identified a novel binding partner of LOXL4, i.e., annexin A2. LOXL4 catalyzes cell surface annexin A2 to achieve a cross-linked multimerization of annexin A2, which in turn prevents the internalization of integrin β-1, resulting in the locking of integrin β-1 on the cell surface. These events enhance the promotion of cancer cell outgrowth.

**Conclusions:**

LOXL4 has a new role in breast cancer progression that occurs *via* an interaction with annexin A2 and integrin β-1 on the cell surface.

## 1 Introduction

Since cancer is a neoplasm and a foreign substance, the inflammation triggered by primary cancer provides cancer cells with a driving force for invasion, which causes them to travel toward their favorite distant organ(s) more quickly. Multiple processes with a variety of signal mechanisms contribute to this inflammation-mediated metastatic progression. For example, we have long studied S100A8/A9, a heterodimer complex of the S100-family proteins S100A8 and S100A9; S100A8/A9 is a pioneer secretory factor for the metastatic progression of cancer cells through the mastery of not only cancer cells themselves but also the cancer microenvironment (1–4). The main suppliers of S100A8/A9 are inflammatory cells including neutrophils (5), monocytes (5), macrophages (5), and myeloid-derived suppressor cells (MDSCs) (5) that gather in a cancer *in situ* microenvironment and in a premetastatic microenvironment in organs that are favored by cancer, such as the lungs. Our efforts have revealed that S100A8/A9 induces the metastatic progression of breast cancer cells upon binding with an S100A8/A9 receptor, i.e., melanoma cell adhesion molecule (MCAM) on cancer cells (3). We were able to verify the binding that activates the MCAM signal pathway, that is, the tumor progression locus 2 (TPL2)-ETS variant transcription factor 4 (ETV4)-zinc finger E-box binding homeobox 1 (ZEB1) axis, resulting in the epithelial-mesenchymal-transition (EMT) and subsequent disseminative invasion and the lung tropic metastasis.

In our more recent research, a question arose: what triggers the marked elevation of S100A8/A9 at the molecular level? Zenz et al. described their interesting observation that in a mouse model, the abrogation of JunB transcription factor in keratinocytes triggered the induction of S100A8/A9, leading to psoriasis-like skin inflammatory disease (6). Thomsen et al. demonstrated that the loss of JunB in prostate cancer cells promotes the expression of S100A8/A9 in the cells’ cancer stroma, which arranges the cancer milieu to a suitable milieu for cancer-invasive progression (7). We speculated that a genetic ablation or downregulation of JunB or the proteinous-functional dysregulation of JunB may contribute to an unfavorable elevation of S100A8/A9 and probably of other soluble inflammatory factors that would function together in a complex manner to directly activate cancer cells themselves and to provide a suitable site for cancer cells to transition toward more aggressive phenotype. To test this idea in breast cancer cells in the present study, we force-delivered a plasmid carrying a mutant JunB that lacks DNA binding ability (JunB ΔDNA) into MDA-MB-231 cells. After an RNAseq analysis of the JunB ΔDNA-overexpressed cells, a single molecule attracted our attention: lysyl oxidase-like 1 (LOXL1) (**Supplementary Figure S1A**).

LOXL1, a secretory soluble protein, is a member of the LOX family (as are LOX, LOXL1, LOXL2, LOXL3, and LOXL4) (8). An accumulation of evidence has revealed that LOX family proteins have an unusual role in many types of cancers’ invasive progression through their catalytic activity, which leads to extracellular remodeling (9). LOX family proteins generally induce the lysyl-oxidation of collagen that leads to fibrillary collagen crosslinking, which in turn activates focal adhesion kinase (FAK) coupled with the cell surface integrins, resulting in a hastening of cancer growth, migration, and invasion. However, the function(s) of LOXes in cancer disease are not limited to extracellular matrix remodeling. Cox et al. reported that LOX plays a crucial role in breast cancer metastasis toward the bone (10). The LOX secreted at bone directly targets osteoclasts to activate them, leading to enhanced bone breakdown, by which bone lesions are formed. The lesions create a premetastatic niche for breast cancer metastasis.

LOX proteins thus appear to have the potential to directly affect cells, probably through cell surface receptor molecules other than the extracellular matrices. In the present study, we considered LOX family proteins other than S100A8/A9 to clarify the significance of LOX family protein(s) in breast cancer progression, since their functions in breast cancer cells have not been fully elucidated.

The results of our experiments and analyses demonstrate that LOXL4 is highly upregulated in triple-negative breast cancer (TNBC) MDA-MB-231 cells. Our efforts unveiled a novel binding and catalyzing partner of LOXL4: the cell surface protein annexin A2. We observed that an abundance of LOXL4 induces the multimerized form of annexin A2 *via* cross-linking, resulting in an inhibition of integrin β-1 internalization, which promotes an integrin β-1-mediated elevation of cancer cell outgrowth. Our discovery of this new mechanism may contribute to an effective therapeutic approach to the prevention of the progression of breast cancer.

## 2 Materials and methods

### 2.1 Cell lines and reagents

The cell line mainly used in this study was MDA-MB-231 (a human triple-negative breast cancer [TNBC] cell line; ATCC, Rockville, MD, USA). We also used another human TNBC cell line, MDA-MB-436 (ATCC), BT-549 (ATCC), and HCC3153, which was kindly provided by Dr. Adi F. Gazdar (Hamon Center for Therapeutic Oncology Research and Department of Pathology, the University of Texas Southwestern Medical Center at Dallas, Dallas, TX, USA). In addition, we used the normal human OUMS-24 fibroblast cell strain that was established by Dr. Masayoshi Namba (11), HEK293T cells (a human embryonic kidney cell line stably expressing the SV40 large T antigen; RIKEN BioResource Center, Tsukuba, Japan), and MCF-7 cells (a human luminal breast cancer cell line; ATCC). All cell lines were cultivated in DMEM/F12 medium (Thermo Fisher Scientific, Waltham, MA) supplemented with 10% fetal bovine serum (FBS). The RGD peptide (RGD [Arg-Gly-Asp] peptide) was purchased from Selleck Chemicals (Houston, TX).

### 2.2 Expression constructs and stable sublines

The mammalian gene expression constructs used in this study were all made using the pIDT-SMART-C-TSC vector (pCMViR-TSC) as the backbone to express the cargo genes at significantly high levels (12). The cDNAs located on the multi-cloning site of the pCMViR-TSC were designed to be expressed in a C-terminal 3Myc-6His-tagged or 3HA-6His-tagged form. The cDNAs encoding GFP, LOXL4 wt, LOXL4 mutCA (see **Supplementary Figure S1E**), and annexin A2 (ANXA2) were inserted into the multi-cloning site of the pCMViR-TSC. Transient transfection of these plasmids into cultured cells was performed using FuGENE-HD (Promega BioSciences, San Luis Obispo, CA) (see **Supplementary Figures S2A, B**).

To obtain stable transformants, we used our original vector named pSAKA-4B (13). A couple of cDNAs encoding GFP, wild-type LOX family members, and LOXL4 mutCA were inserted into the multi-cloning site of pSAKA-4B. The clones stably overexpressing these genes were established by a convenient electroporation gene delivery method and subsequent selection with puromycin at 20 μg/mL. MDA-MB-231 LOXL4 knock-out (KO) sublines were generated according to the CRISPR/Cas9 method (see **Supplementary Figure S1C**) with the gRNA2 (CRISPR sequence: ACCAGTGCGGGTCTAATGGC) expression plasmid that expresses Cas9 at the same time (Thermo Fisher Scientific).

### 2.3 Cell assays

Cells (2 × 10^3^ cells) were seeded in 96-well culture plates. A CellTiter 96^®^ AQueous One Solution Cell Proliferation Assay (MTS) (Promega Biosciences) was used for the assessment of cell proliferation. Cell migration and invasion were evaluated by a Boyden chamber assay with a Matrigel-coated (for invasion) or non-coated (for migration) transwell membrane filter insert (pore size, 8 μm) in a 24-well plate (BD Biosciences, Franklin Lakes, NJ). Cells were starved by low-serum (0.5% FBS) DMEM/F12 medium prior to the assay. Cells (2 × 10^4^ cells/insert) were seeded with low-serum (0.5% FBS) DMEM/F12 medium on the upper chamber, and the lower chamber was filled with DMEM/F12 medium containing 10% FBS. After incubation for 24 hr, cells that passed through the filter were counted by staining with hematoxylin and eosin (H&E) solution. Migrated or invaded cells were imaged under a microscope (BZ-9000; Keyence, Tokyo) and quantified by cell counting in five non-overlapping fields at ×100 magnification. The numbers of migrated or invaded cells are presented as the average from three independent experiments.

### 2.4 Real-time quantitative PCR

Total RNA was extracted from the cells using ISOGEN II Isolation Reagent (Nippon Gene, Tokyo). Reverse transcription was then performed using ReverTraAce qPCR RT Master Mix with gDNA Remover (Toyobo, Osaka, Japan). A real-time polymerase chain reaction (PCR) was performed using FastStart SYBR^®^ Green Master Mix (Roche Applied Science, Penzberg, Germany) with specific primers on a StepOnePlus™ Realtime PCR system (Applied Biosystems, Foster City, CA). The following forward and reverse primer pairs (5′ to 3′) were used: *TBP* (forward: GAACATCATGGATCAGAACAACA; reverse: ATAGGGATTCCGGGAGT), *LOX* (forward: TGAAAAACCAAGGGACATCAG; reverse: GGCATCAAGCAGGTCATAG), *LOXL1* (forward: ACCAGGGCACAGCAGACTT; reverse: GTGGCTGCATCCAGTAGGTC), *LOXL2* (forward: CTACGTGGAGGCCAAGTCC; reverse: CGTTGCCAGTACAGTGGAGA), *LOXL3* (forward: CAACAGGAGGTTTGAACGCTAC; reverse: GCTGACATGGGTTTCTTGGTAA), *LOXL4* (forward: TGCCGCTGCAAGTATGATG; reverse: TGTTCCTGAGACGCTGTTCC).

### 2.5 Knockdown experiments

Human annexin A2 siRNA (siANXA2, ID: s1383), integrin β-1 siRNA (siITGB1, ID: s7575), and control siRNA (silencer select negative control No. 1 siRNA) were purchased from Thermo Fisher Scientific, and the siRNAs were transfected by Lipofectamine RNAiMax (Thermo Fisher Scientific) with the final concentration of 30 nM. After incubation for 72 hr, transfected cells were subjected to cell assays.

### 2.6 Western blotting

The western blotting assay was performed under conventional conditions. The antibodies used were as follows: rabbit anti-human LOX monoclonal antibody (Cell Signaling Technology, Beverly, MA), rabbit anti-human LOXL1 polyclonal antibody (Proteintech Japan, Tokyo), mouse anti-human LOXL4 monoclonal antibody (Santa Cruz Biotech, Santa Cruz, CA), rabbit anti-human annexin A2 monoclonal antibody (Cell Signaling Technology), rabbit anti-human integrin β-1 polyclonal antibody (Proteintech Japan), mouse anti-tubulin monoclonal antibody (Proteintech Japan), mouse anti-HA tag antibody (Cell Signaling Technology), mouse anti-Myc antibody (Cell Signaling Technology), and goat anti-GST antibody (Cytiva, Marlborough, MA).

### 2.7 Enzymatic reaction in a cell-free system

The glutathione transferase (GST)-fusion annexin A2 (GST-annexin A2) was prepared in an *Escherichia coli* (*E. coli*) expression system using pGEX6P1 vector (GE Healthcare, Chicago, IL) as described (14). The HA-tagged LOXL4 wt (wildtype) and mutant (mut)CA proteins were prepared by a pull-down method using anti-HA tag agarose beads (Sigma Aldrich) from their overexpressed HEK293T cell lysates. The *in vitro* LOXL4-mediated oxidation reaction with GST-annexin A2 was performed according as described by Kim et al. (15) with a small modification. For the evaluation of the LOXL4-mediated oligomerization of annexin A2, we mixed GST-annexin A2 (10 μg) and the pulled-down LOXL4 specimens (each 2.5 μg) in a non-amine-based reaction buffer (50 mM HEPES/pH 7.4, 150 mM KCl, 1% glycerol, 7.5 μM CuSO_4_, and protease inhibitor cocktail (Roche Applied Science) to a total volume of 100 μL in a test tube. The mixed specimens were then incubated at 37°C for 24 hr.

For the assessment of the LOXL4-mediated aldehyde(s) formation of annexin A2 as a result of its oxidation for lysine residue(s), we used biotin-conjugated hydrazide (Biotin-PEG4-Hydrazide, Tokyo Chemical Industry, Tokyo), which specifically reacts with aldehyde. The chemical was added to the reactants that were prepared alternatively according to the above-described method with the following difference: the incubation time of 6 hr after the liquid pH was adjusted to ~6.0 with the other non-amine-based buffer MES (1 M/pH 6.0) that gave the final concentration of MES 500 mM. The mixture was then incubated at 37°C for 2 hr, and the reaction was blocked by the further addition of the amine-based Tris buffer. The biotin-labeled products were finally collected by streptavidin-conjugated agarose beads (Thermo Fisher Scientific).

### 2.8 Immunocytochemistry of live cells

The live cells cultured on type IV collagen-coated cover glasses were treated with rabbit anti-human annexin A2 polyclonal antibody (Proteintech Japan) or rabbit anti-human integrin β-1 polyclonal antibody (Proteintech Japan) without fixation for 60 min. After the treated live cells were washed with the conditioned medium, they were fixed with 4% paraformaldehyde (PFA) and stained with the fluorescence-labeled anti-rabbit IgG secondary antibody (goat anti-rabbit IgG [H+L] and the highly cross-adsorbed secondary antibody, Alexa Fluor™ 594, Thermo Fisher Scientific).

### 2.9 Animal experiments

The animal experimental protocols were approved by the Animal Experiment Committee of Okayama University (approval no. OKU-2020001). All of the mouse procedures and euthanasia, including cell transplantations, were done painlessly or with the mouse under anesthesia according to the strict guidelines of the University’s Experimental Animal Committee. Cells of interest (5 × 10^5^ cells) suspended in Matrigel were injected into the mammary fat pads of 7-week-old Balb/c nude mice (Charles River Laboratories, Yokohama, Japan). The tumor volume was measured every 3 days as ½ × (longest dia.) × (shortest dia.)^2^.

The frozen sections prepared from every resected-frozen-tissue block were subjected to immunofluorescence staining under conventional conditions for immunohistochemistry. The primary antibodies used were rat anti-CD31 monoclonal antibody (BD Biosciences, San Jose, CA) and rabbit anti-COL1A1 monoclonal antibody (Cell Signaling Technology). The tissue-bound primary antibodies were detected by the secondary antibody, goat anti-rat or -rabbit IgG [H+L], and the highly cross-adsorbed secondary antibody, Alexa Fluor™ 594 (Thermo Fisher Scientific). The staining with SYBR^®^ Green I (Thermo Fisher Scientific) detected the tumor-cell nuclei.

### 2.10 Statistical analysis

Each experiment was repeated three times, and the resulting raw data were statistically analyzed. The calculated values are the mean ± standard deviation (SD) or mean ± standard error of the mean (SEM). One-way analysis of variance (ANOVA) was performed for the comparative evaluation of more than two groups. When the ANOVA shows a significant difference, the Bonferroni procedure was used as a *post hoc* test. Probability (p)-values <0.05 were considered statistically significant.

## 3 Results

### 3.1 LOXL4 promotes breast cancer cell outgrowth

**Figure 1:**
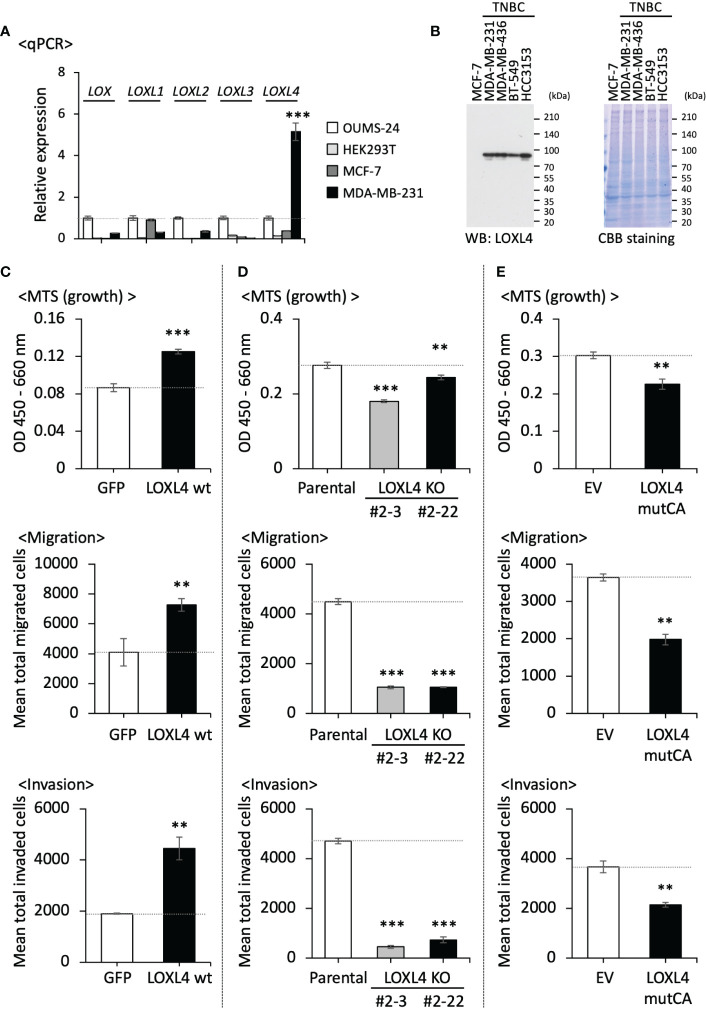
The beneficial roles of LOXL4 in the growth, migration, and invasion of triple-negative breast cancer (TNBC) cells *in vitro*. **(A)**: A real-time qPCR was performed to determine the endogenous levels of *LOX* family mRNAs in the indicated cells. *TBP* mRNA was used as a control for the analysis. **(B)** The intrinsically expressed LOXL4 protein in the indicated cell lines was also detected using the WB procedure. **(C, E)** We evaluated the growth (*upper*), migration (*middle*), and invasion (*lower*) abilities of the established MDA-MB-231 sublines with the stable overexpression of the wildtype of LOXL4 (LOXL4 wt subline) **(C)**, the genetic ablation of the intrinsic *LOXL4* gene (LOXL4 KO #2-3 and #2-22 sublines) **(D)**, and the stable overexpression of mutant LOXL4 losing the catalytic activity (LOXL4 mutCA subline) **(E)**. Data are mean ± SD. **p<0.01, ***p<0.001.

Our qPCR-based analysis of the LOX family members’ profiles revealed that LOXL4 is the most highly expressed gene among the LOX family members in an invasive TNBC line, i.e., MDA-MB-231 cells but not a non-invasive luminal cell line, MCF7 cells (**Figure 1A**). The increased level of LOXL4 expression in MDA-MB-231 cells was confirmed at protein levels, and it was consistently observed in the other three sorts of TNBC cell lines (**Figure 1B**). To clarify the significance of the elevated LOXL4 expression in TNBCs, we established a stable transformant overexpressing wild-type (wt) LOXL4 from MDA-MB-231 cells (**Supplementary Figure S1B**), and we evaluated its cancer-relevant activities. The subline showed markedly elevated cell growth, migration, and invasion compared to the control GFP-overexpressed MDA-MB-231 cells (**Figure 1C**). To further examine this result, we genetically ablated the intrinsic *LOXL4* gene by applying the CRISPR/Cas9 method, which resulted in the establishment of two sublines, MDA-MB-231-LOXL4 KO #2-3 and #2-22 (**Supplementary Figures S1C, D**). The cellular behaviors, growth, migration, and invasion were all significantly dampened in both of the KO sublines (**Figure 1D**).

We then created the catalysis-deficient mutant of LOXL4 (LOXL4 mutCA) in order to analyze the enzymatic role of LOXL4 in cellular behaviors. According to Oldfield et al. (16), the essential amino acid 622-histidine (H) as the catalytic base in LOX is well-conserved among LOX family members and was replaced with aspartic acid (D) (**Supplementary Figure S1E**). The important amino acids for trapping copper ion (Cu^2+^) in LOXL4, i.e., 611H, 613H, and 615H, each of which is also well-conserved in all LOX family members, were all additionally replaced with alanine (A) since Cu^2+^ binding also contributes to the activation of LOXes (17). With the use of this construct, the stable cell subline that overexpressed LOXL4 mutCA was established from MDA-MB-231 cells, and the expression of the integrated foreign gene was confirmed (**Supplementary Figure S1F**). In agreement with the results obtained with the KO cells, the cellular activities of growth, migration, and invasion were all significantly lowered (**Figure 1E**).

**Figure 2:**
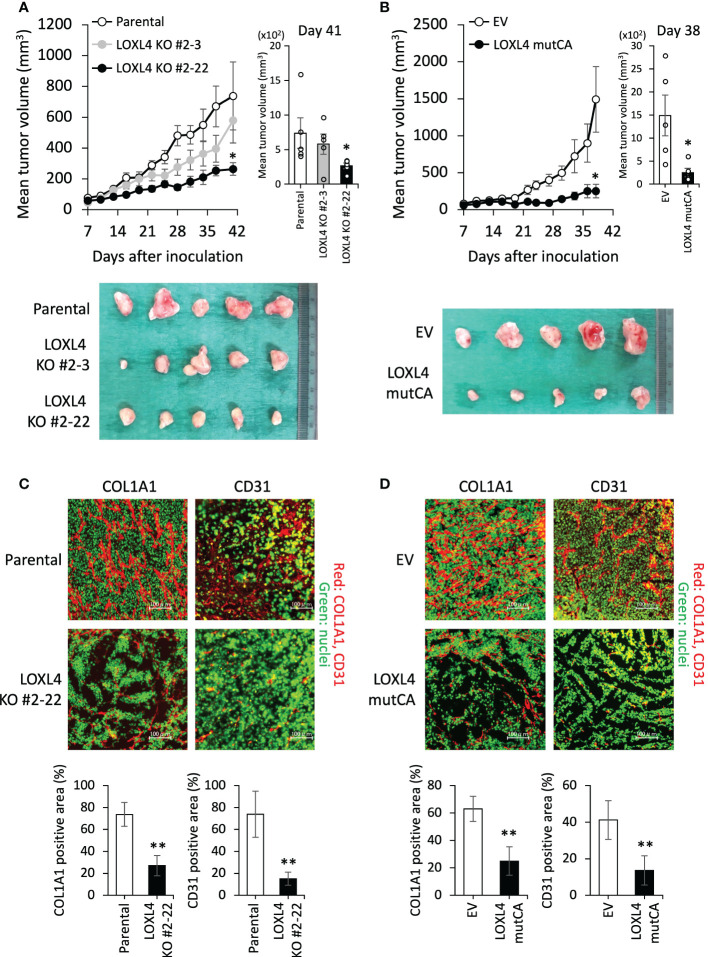
The effects of the genetic deletion and catalytic deficiency of LOXL4 on cancer cell growth *in vivo*. **(A, B)**: In the orthotopic xenograft mouse model, a tumor growth curve was established after injection of the indicated cells **(A)**: parental cells and LOXL4 KO sublines, **(B)**: control empty vector (EV) and LOXL4 mutCA sublines). The tumor diameters were measured (*upper* (line graphs of chronological evaluation: *left*, bar graphs at the end of assessment: *right*)), and photographs of the dissected tumors were taken (*lower*). Data are mean ± SEM (n=5) for animal experiments. *p<0.05. **(C, D)**: The resected-tumor sections were immunofluorescence-stained by the indicated antibodies **(C)**: parental cells and LOXL4 KO sublines, **(D)**: control empty vector (EV) and LOXL4 mutCA sublines). The SYBR^®^ Green I staining detected nuclei. The bars in the images represent 100 μm. The staining images were exhibited on the upper side, and their quantified data were displayed on the lower side. Data are mean ± SD. **p<0.01.

We then extended our approach to an *in vivo* experiment to investigate the role of LOXL4 in cancer outgrowth in the orthotopic xenograft mouse model. As shown in **Figure 2A**, the KO cell-derived cancer growth was predominantly stalled in both sublines compared to the parental cell line’s growth (**Figure 2A**). In line with this, the growth rate of the LOXL4 mutCA subline was also very slow compared to that of the control stable subline that possessed the control empty vector (EV) (**Figure 2B**). These results suggest that LOXL4 promotes cancer cell outgrowth *via* its enzymatic activity. Owing to the firm involvement of LOX family proteins in fashioning cancer preferential milieu *via* remodeling of extracellular matrices *via* collagen cross-linking, which gives rise to a plethora of stable collagen (18), and thereby facilitating cancer-associated angiogenesis (19), the status of collagen presence and angiogenesis was studied in the resected tumors as imaged in the lower panels of **Figures 2A, B**. Interestingly, the staining levels of COL1A1, a significant component of cancer-associated collagens, and CD31, a representative endothelial cell marker, were reduced substantially in the LOXL4 KO cell-derived tumors and the LOXL4 mutCA subline-derived tumors (**Figures 2C, D**).

### 3.2 Identification of the cell surface annexin A2 as a binding receptor of LOXL4

The pathological significance of the extracellular remodeling regulated by LOXes *via* their enzymatic activities in cancer progression has been described before (9), and in the present study, we thus attempted to determine the autocrine potential of LOXL4 in cancer cells, since another LOX family member acts directly on the cell surface and activates the sensitized cells without extracellular matrices (10). To explore cell-surface LOXL4 binding protein(s) in MDA-MB-231 cells, we collected the conditioned medium of the C-terminal HA-tagged LOXL4-overexpressed HEK293T cells, poured it into MDA-MB-231 culture, and collected the cultured cell pellets. The cell lysate was then subjected to immunoprecipitation with anti-HA-tag Ab-conjugated beads and a mass spectrometric analysis (**Supplementary Figure S2A**). This approach identified an interesting molecule, annexin A2, which is firmly associated with cancer metastatic progression in a plethora of cancer types including breast cancers (20). The putative annexin A2-like protein detected herein is a pseudogene of annexin A2. The binding of LOXL4 and annexin A2 was then confirmed by the immunoprecipitation approach (**Supplementary Figure S2B**), and the interaction was specific to LOXL4 among the LOX family members.

Our next approach, i.e., the comprehensive identification of plasma membrane proteins by the method called ‘cell surface biotin labeling-pull down of the biotin-labeled proteins-MS/MS shotgun analysis of the precipitated proteins,’ also achieved identified annexin A2, which was enriched in MDA-MB-231 parental cells (**Supplementary Figure S2C**). Integrin β-1 also piqued our interest. The proteins, annexin A2 and integrin β-1 were not identified in the LOXL4 KO or mutCA sublines. In light of this interesting result, we suspected that the secreted LOXL4 may bind with cell-surface annexin A2 and catalyze it, resulting in integrin β-1 enrichment on the cell surface, which could work to promote the progression of breast cancer.

**Figure 3:**
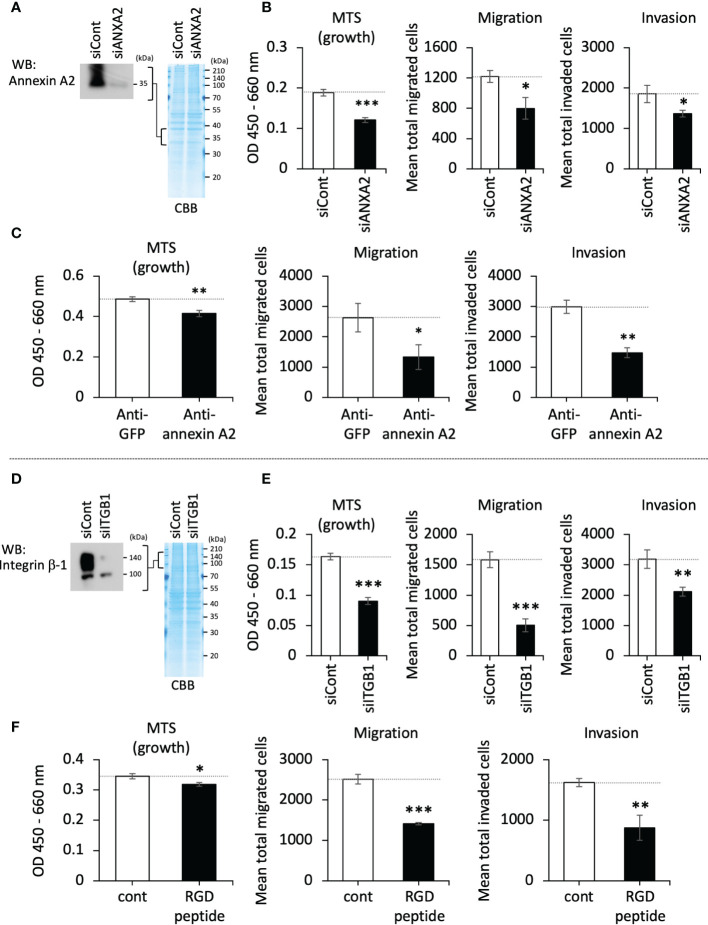
The roles of cell-surface annexin A2 and integrin β-1 on cellular growth, migration, and invasion *in vitro*. **(A)**: The siRNA-mediated lowered expression of the intrinsic annexin A2 in MDA-MB-231 cells was confirmed by western blotting (WB). **(B)**: The growth, migration, and invasion of annexin A2 siRNA (siANXA2)-treated cells. **(C)**: MDA-MB-231 cells were treated with annexin A2 antibody (anti-annexin A2, final conc. 25 μg/mL) to block the cell-surface annexin A2. The treated cells’ growth, migration, and invasion were then evaluated. Green fluorescence protein (GFP) antibody (anti-GFP) developed by our laboratory (final conc. 25 μg/mL) was used as a negative control. **(D)**: MDA-MB-231 cells were treated with integrin β-1 siRNA (siITGB1), and the reduced expression of the protein was confirmed by WB. **(E)**: The siRNA-treated cells were evaluated as described for panel **(C) (F)**: RGD peptide (500 μg/mL) was used to inhibit the cell-surface integrins, and its effects on cell growth, migration, and invasion were evaluated. Data are mean ± SD except for those in panels **(A, D)** *p<0.05, **p<0.01, ***p<0.001.

The binding of annexin A2 with integrin β-1 has been reported; the interaction of annexin A2 and integrin β-1 on the cell surface functions to achieve an active internalization of integrin β-1, leading to a downregulation of adhesion activity in intestinal epithelial cells (21). Bearing in mind this reported event, we hypothesized that LOXL4 prevents this machinery as an adept cancer trait. To test this hypothesis, we first tried to determine the effects of annexin A2 and integrin β-1 on cancer cell behaviors *in vitro*. The downregulation of intrinsic annexin A2 in MDA-MB-231 cells (**Figure 3A**) force-weakened the cells’ growth, migration, and invasion (**Figure 3B**). Extracellularly added annexin A2 antibody also inhibited these cellular events (**Figure 3C**). In the case of integrin β-1, we observed reactions that were similar to those obtained with annexin A2 siRNA (siANXA2) or the antibody-treated cells when integrin β-1 siRNA (siITGB1) was used (**Figures 3D, E**) or when the integrin inhibitor RGD peptide was used (**Figure 3F**). These results indicate functional significance of the cell-surface annexin A2 and integrin β-1 in breast cancer activities that is associated with disseminative progression *in vitro*.

### 3.3 Interplay among LOXL4, annexin A2, and integrin β-1

**Figure 4:**
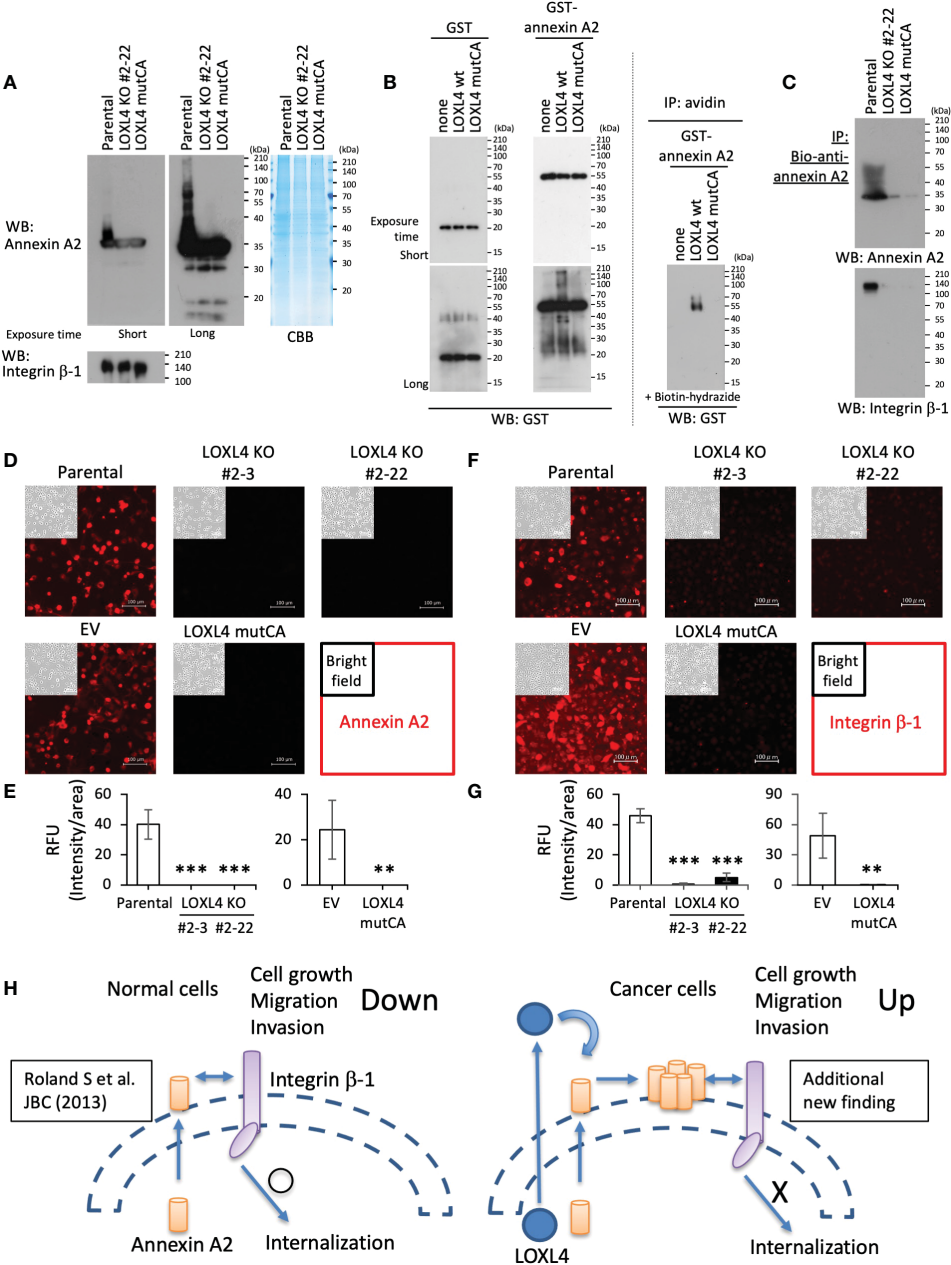
LOXL4-mediated regulation of the cell-surface locations and functions of annexin A2 and integrin β-1. **(A)**: The indicated cells were lysed and subjected to WB for the evaluation of their intrinsic levels and states of annexin A2 and integrin β-1. **(B)**: The pulled-down LOXL4 wt or mutCA was mixed with the prepared recombinant proteins (GST or GST-annexin A2) according to the indicated formula, and the mixed solutions were then incubated at 37°C for 24 hr (*left*). The reactants were then subjected to WB with the GST antibody. The same reactants were also prepared alternatively, and the reaction was stopped at 6 hr; the reactants were further subjected to a biotin-conjugated hydrazide reaction, and the biotin-labeled GST-annexin A2 was collected by streptavidin-conjugated agarose beads (*right*). **(C)**: The indicated live cells were treated with the biotinylated (bio)-annexin A2 antibody to selectively capture the cell-surface annexin A2 of the individual cells. The captured annexin A2 was collected by the pull-down method, and the precipitates were further subjected to WB with the indicated antibodies (see the protocol shown in **Supplementary Figure S2D**). **(D, E)**: The indicated live cells were treated with annexin A2 antibody to detect the cell-surface annexin A2. Representative images **(D)** are shown and their signals were quantified. RFU: relative fluorescent unit. **(F, G)**: The indicated live cells were also treated with integrin β-1 antibody to detect the cell-surface integrin β-1. Representative images **(F)** are shown, and are their quantified data **(G)**. **(H)**: Schematic diagram of the molecular interplay among LOXL4, annexin A2, and integrin β-1. Data are mean ± SD, **p<0.01, ***p<0.001.

Bearing in mind the above findings, we next investigated the interplay among LOXL4, annexin A2, and integrin β-1 according to our hypothesis. Of note, the annexin A2 in the parental cells appeared as ladder bands toward high molecular weights, but this did not occur in protein bands from either the LOXL4 KO or mutCA sublines even with the extended exposure time (**Figure 4A**). The ladder formation was also observed in the *in vitro* cell-free system in which recombinant annexin A2 was mixed with the immunoprecipitated foreign LOXL4 wt protein that is transiently overexpressed in HEK293T cells (**Figure 4B**, left). Consistent with this result, the ladder band was not growing when annexin A2 was mixed with the immunoprecipitated foreign LOXL4 mutCA protein. We also confirmed that the LOXL4 wt-specific ladder formation was concomitant with the aldehyde(s)’ formation of annexin A2 (**Figure 4B**, right). These results indicate that the ladder formation of annexin A2 was attributable to cross-linking by LOXL4-mediated catalytic oxidation, which resulted in the multimerization of annexin A2.

To investigate the significance of the LOXL4-mediated multimerization of annexin A2 on cell surface integrin β-1, we further examined the interaction state of annexin A2 with integrin β-1 and their occupation rate on the cell surface in the parental MDA-MB-231 cells and their variant sublines. With the protocol shown in **Supplementary Figure S2D**, we observed that the multimerized annexin A2 was effectively collected from the cell surface (**Figure 4C**), and where the binding between annexin A2 and integrin β-1 was maintained at a significant level in parental cells but not in the LOXL4 KO and mutCA sublines. In line with this result, we detected the cell-surface annexin A2 (**Figures 4D, E**) and integrin β-1 (**Figures 4F, G**) in the parental cells but not in the LOXL4 KO and mutCA sublines.

Cumulatively, these findings suggest that MDA-MB-231 cells are adept at avoiding the negative machinery of cell growth, migration, and invasion caused by the annexin A2-mediated internalization of integrin β-1 (**Figure 4H**). The abundance of LOXL4 in MDA-MB-231 cells targets cell-surface annexin A2 in an autocrine manner to cross-link it, resulting in the multimerization of annexin A2 that in turn firmly locks integrin β-1 on the cell surface by preventing the internalization of the protein. Finally, the enriched integrin β-1 on the cell surface enables cancer cells to activate their cellular behaviors related to cancer outgrowth.

## 4 Discussion

Although the contributions of LOX family proteins to cancer progression have long been recognized by scientists, the precise details of these contributions have not been established. In general, LOXes are secreted and help catalyze the lysyl oxidation of components of the extracellular matrix such as collagen, which leads to cross-linking, thus providing stiffness of the matrix as a cellular anchor (19). In a cancer milieu, an unfavorable enzymatic activation or an elevated level of LOXes is associated with enhanced fibrosis, which hastens cancer cell growth and invasion through the activation of the integrins’ coupled FAK of cancer cells (18). In addition, some unidentified functions of LOXes that are different from the cross-linking of collagen have been reported. Cox et al. revealed that a LOX abundantly produced and secreted by breast cancer cells directly sticks to osteoclasts and activates them, leading to bone destruction and resulting in the building of cancer’s most suitable premetastatic space (10).

It thus seems that LOXes have other target molecule(s) on the cell surface that are distinct from extracellular matrices. In the present study, we decided to focus on LOXL4 among the LOX family proteins because of the unusual increase in LOXL4 (among all of the LOX family members) in MDA-MB-231 breast cancer cells, which is consistent with those in other TNBC cell lines; in addition, this finding is in agreement with the results described by Choi et al. (22). This may be a reasonable event, since LOXL4 is known to be upregulated by a hypoxic condition (23), transforming growth factor (TGF)-β (24), and an EMT-core transcription factor ZEB1 (25), all of which are associated with the malignant conversion of breast cancers. Extracellular matrix remodeling in the cancer milieu by LOX family members is a well-known function of LOX proteins, and we, therefore, sought to identify the still-unidentified role of upregulated LOXL4 in breast cancer. Our efforts revealed a new target of LOXL4: cell-surface annexin A2. The LOXL4-mediated multimerization of annexin A2 in a catalytic cross-linking manner prevented the internalization of integrin β-1, whereby cellular growth, migration, and invasion were all enhanced due to the sustained presence of integrin β-1 on the cell surface. The novel mechanism of LOXL4 that was revealed in the present study may broaden the research concerning LOX-family proteins in cancer biology.

It should be noted that the function of LOXL4 in the progression of breast cancer is a contentious topic. Our present findings are in good agreement with those of a study reported by Yin et al. (26), in which *in vitro* cell growth and migration evaluations as well as an *in vivo* tumor outgrowth evaluation using the same MDA-MB-231 cells revealed that the induction of LOXL4 was involved in the progression of breast cancer metastases. In contrast, Choi et al. reported a tumor-suppressive function of LOXL4 in MDA-MB-231 cells (22). This discrepancy in findings may be due to different culture conditions used for the MDA-MB-231 cells, which often induce a characteristic alteration such as the amplification of a specific variant population whose traits will differ from those of the original population. For example, the luminal breast cancer cell line MCF-7 has a derived variant, MCF-7-V, that displays more robust estrogen receptor (ER) α responses (27). Another reason for the discrepancy may be the presence of splice variants of LOXL4 whose expression patterns will differ if the variants appeared under different culture conditions or protocols. Sebban et al. revealed that LOXL4 is alternatively spliced in cancer cells (28). When they force-delivered the spliced variants (type 1 and type 2) of LOXL4 into MDA-MB-231 cells to overexpress them in a comparison with the full-length form, they observed that both variants but not the full-length form promoted metastatic cellular events in both *in vitro* and *in vivo* experimental settings. Conceivably, functional dimensions of LOXL4 such as secretion, catalysis, and the binding to annexin A2 will vary in accord with the splicing event of LOXL4, which may reflect our observation that the genetic ablation of intrinsic LOXL4 or mutant LOXL4 losing catalytic activity stalls the outgrowth of breast cancer. We observed that the wildtype of the LOXL4-overexpressing MDA-MB-231 subline showed a trend of dampened cell growth in the orthotopic xenograft mouse model compared to the control GFP-overexpressed cells (**Supplementary Figure S2E**). The unexpected *in vivo* result is consistent in part with the result described by Choi et al. (22). We are thus planning to further investigate the possibility of new LOXL4-targeting molecules corresponding to the spliced forms in a comprehensive manner.

Annexin A2 may not be the only novel binding substrate of LOXL4. One of our earlier studies of oropharyngeal squamous cell carcinomas (SCCs) revealed the elevation of procollagen-lysine, i.e., 2-oxoglutarate 5-dioxygenase 2 (PLOD2), which is a secretory enzyme like LOXes that is well known to catalyze the lysyl-hydroxylation of collagen attributed to its maturation; the increased level of the protein is associated with metastasis *via* the recognition of integrin β-1 and the hydroxylation of it as a novel substrate to lead to the stabilization of integrin β-1 on the cell surface (29). Thus, it will be standard for the soluble secretory enzymes that catalyze collagens to have multiple substrates other than collagens. Besides annexin A2, we also fortuitously exploited to identify the additional LOXL4 substrate protein on the cancer cell surface, which is conceivable to act to dampen NK cell activation (**our unpubl. data**). The protein expression was lowered in the two LOXL4 KO cells (#2-3 and #2-22) in a consistent manner, but with a little difference in expression between the two. Contemplating the possibility of the protein workable in cell growth regulation may explain why the LOXL4 KO #2-3 is the most efficient in stalling cell growth *in vitro* but is oppositely inefficient *in vivo*. Our following study will define the relationship between LOXL4 and the newly identified candidate substrate on the cell surface in cancer outgrowth *in vitro* and *in vivo*.

It is also necessary to discuss the unusual role of annexin A2 in cancer progression. Annexin A2 has been reported to be highly upregulated in the cells of multiple types of cancer (including breast cancer), with an especially higher expression in the invasive triple-negative MDA-MB-231 cell line compared to the noninvasive luminal cell line MCF-7 (20, 30). Our previous investigation demonstrated that annexin A2 helps reseal the injured plasma membrane, which accelerates cell migration and the invasion of breast cancer cells since the plasma membrane is exposed to severe physical stress that compromises cell dissemination movements (31). In line with that finding, the binding of annexin A2 to actin filaments at the C-terminal site contributes to cancer cells’ spread *via* the regulation of the dynamic remodeling of the actin cytoskeleton (20). The intracellular annexin A2 also induces the EMT through the activation of the STAT3 transcription factor by direct binding to it (20). Annexin A2 is also located on the cell surface when phosphorylated at tyrosine 23 by the proto-oncogene tyrosine-protein kinase Src in cancer cells (32). It was also reported that in pancreatic ductal adenocarcinoma (PDA) cells, cell-surface annexin A2 promotes cancer cell invasion and metastasis upon binding with the extracellular matrix component tenascin C (TNC) (33). Cell-surface annexin A2 is also required in the conversion of plasminogen to plasmin in MDA-MB-231 cells, which gives rise to increased invasion in the cancer cells (34). Contrary to these findings, cell-surface annexin A2 seems to have a negative aspect on cancer progression. Rankin et al. reported that in intestinal epithelial cells, annexin A2 on the cell surface actively works to internalize integrin β-1 *via* their direct binding, whereby the adhesion activity is severely limited (21). The underlying mechanism of this would work against cancer progression. In our investigation of this negative machinery, we first observed that the TNBCs were adept at avoiding the machinery by exploiting LOXL4; that is, the LOXL4-mediated multimerization of annexin A2 locks the integrin β-1 on the cell surface. Our efforts also identified integrin α-6 as another molecule that accumulated on the cell surface when normal LOXL4 was abundantly expressed (**Supplementary Figure S2C**). The heterodimer complex combination of integrin α-6 and integrin β-1has been reported to promote cancer metastatic outgrowth in MDA-MB-231 cells (35).

Besides the active working of the LOXL4-mediated abundant COL1A1 as a suitable scaffold for the cell migration and growth of the cancer-associated endothelial cells as well as those of cancer cells, the cancer cell surface annexin A2 force-locked by LOXL4 may be involved in the enhanced angiogenesis event (**Figures 2C, D**). Chaudhary et al. reported that exosomal-annexin A2 promotes angiogenesis in TNBC cells (36). In light of the facts that ELISA detects the intact exosomal-annexin A2 in the sera of the TNBC-bearing patients (36), and anti-annexin A2 antibody inhibits angiogenesis in mouse breast cancer-derived tumor *in vivo* (37), it may be reasonable to consider that the cancer cell surface annexin A2 fixed by the help of LOXL4 also resides on the exosomal surface even after the secretion as exosome and it acts to make grow angiogenesis. Interestingly, exosomal-annexin A2 also contributes to elicit cancer promotable macrophages (38). Considering the pleiotropic role of annexin A2 in cancer progression, we speculate that the novel LOXL4-mediated mechanism identified herein (i.e., a LOXL4-annexin A2-integrin α6/integrin β-1 axis) plus the already reported multiple means of annexin A2 are exerted together simultaneously or separately in not only specific cellular contexts such as proliferation, the EMT, migrative invasion, survival, and adhesion, but also cancer extracellular milieus such as matrices’ remodeling, angiogenesis, and macrophage-mediated immune tolerance, through the processes of cancer metastatic progression.

The relationship between LOXL4 and S100A8/A9 in breast cancer progression remains to be discussed. Our previous efforts showed a pivotal role of the S100A8/A9-MCAM-TPL2-ETV4-ZEB1 pathway which we identified in metastatic progression in TNBCs. It is also of interest that ZEB1 induces an increase in the expression of both LOXL4 (25) and annexin A2 (27) in breast cancer cells, and ZEB1 thus has the potential to naturally enhance the mechanism of LOXL4 in TNBCs upon S100A8/A9 stimulation. It has been reported that LOXL4 monoclonal antibody (LOXL4-mAb) effectively prevents the growth of head and neck squamous cell carcinoma (HNSCC) cells *in vivo* (39). Considering the past and present findings together, we suggest that our developed S100A8/A9-mAb (Ab45) (40) combined with the LOXL4-mAb will be more effective than any single inhibitor for the prevention of breast cancer outgrowth. Very recently, Ramos et al. reported about LOXes for their potential as biomarkers or therapeutic targets through a bioinformatic analysis, arguing that LOX, LOXL1, and LOXL2 will be highly beneficial molecules (41). The suggestion opens up an exciting possibility of mutual functions of these molecules besides LOXL4 in a compensated manner to one another or different operations in part through the process of progression regulatory maze in breast cancers, where the presence of several heterogeneous types of cancer cells and normal plural cells are altered for their proportion in cancer milieu. Single-cell RNA-Seq analysis is further required to clarify the problem, and therefore the subject is our next theme.

## Data availability statement

The original contributions presented in the study are publicly available. This data can be found here: https://www.ddbj.nig.ac.jp/dra/index.html, DRA016031.

## Ethics statement

The animal study was reviewed and approved by OKU-2020001.

## Author contributions

NK designed the study, performed (with NT) most of the experiments, and analyzed the data. RK performed the RNAseq analysis. YC made stable cell lines expressing the LOX family and performed an invasion assay. YG, FJ, K-IY, and HM performed cell assays in part. IR, IS, and JZ helped confirm several *in vitro* results. AY, FK, and ST assisted with the data analysis. YI performed the real-time qPCR analysis. MS performed the plasmid construction, immunoprecipitation, and Western blot analysis with the assistance of NK and NT. NK and MS wrote the manuscript. MS designed and supervised the project and reviewed and edited the manuscript. All authors contributed to the article and approved the submitted version.
