# Supplementary material for: Lysyl oxidase-like 4 exerts an atypical role in breast cancer progression that is dependent on the enzymatic activity that targets the cell-surface annexin A2
Source: Front Oncol. 2023 Apr 4;13:1142907. doi: 10.3389/fonc.2023.1142907 (PMC10114587; doi:10.3389/fonc.2023.1142907)
Supplement: Supplementary file 1 [file DataSheet_1.pdf]

Supplementary Material

Lysyl oxidase-like 4 exerts an atypical role in breast cancer progression that is dependent on the enzymatic activity that targets the cell-surface annexin A2

Ni Luh Gede Yoni Komalasari[1,4], Nahoko Tomonobu[1], Rie Kinoshita[1], Youyi Chen[2], Yoshihiko Sakaguchi[3], Yuma Gohara[1], Fan Jiang[1], Ken-ichi Yamamoto[1], Hitoshi Murata[1], I Made Winarsa Ruma[4], I Wayan Sumardika[4], Jin Zhou[5], Akira Yamauchi[6], Futoshi Kuribayashi[6], Yusuke Inoue[7], Shinichi Toyooka[8], and Masakiyo Sakaguchi[1]*

[1]Department of Cell Biology, Okayama University Graduate School of Medicine, Dentistry and Pharmaceutical Sciences, Okayama, Okayama, Japan

[2]Department of General Surgery & Bio-Bank of General Surgery, The Fourth Affiliated Hospital of Harbin Medical University, Harbin, Heilongjiang, China

[3]Department of Microbiology, Kitasato University School of Medicine, Sagamihara, Kanagawa, Japan

[4]Faculty of Medicine, Udayana University, Denpasar, Bali, Indonesia

[5]Medical Oncology Department of Gastrointestinal Tumors, Liaoning Cancer Hospital & Institute, Cancer Hospital of Dalian University of Technology, Shenyang, Liaoning, China

[6]Department of Biochemistry, Kawasaki Medical School, Kurashiki, Okayama, Japan

[7]Faculty of Science and Technology, Division of Molecular Science, Gunma University, Kiryu, Gunma, Japan

[8]Department of General Thoracic Surgery and Breast and Endocrinological Surgery, Okayama University Graduate School of Medicine, Dentistry and Pharmaceutical Sciences, Okayama, Okayama, Japan

***Correspondence:** Dr. Masakiyo Sakaguchi, Department of Cell Biology, Okayama University Graduate School of Medicine, Dentistry and Pharmaceutical Sciences, 2-5-1 Shikata-cho, Kita-ku, Okayama-shi, Okayama 700-8558, Japan.

Tel.: +81-86-235-7395, Fax: +81-86-235-7400

Email: masa-s@md.okayama-u.ac.jp

SUPPLEMENTARY FIGURES

Suppl. Fig. S1

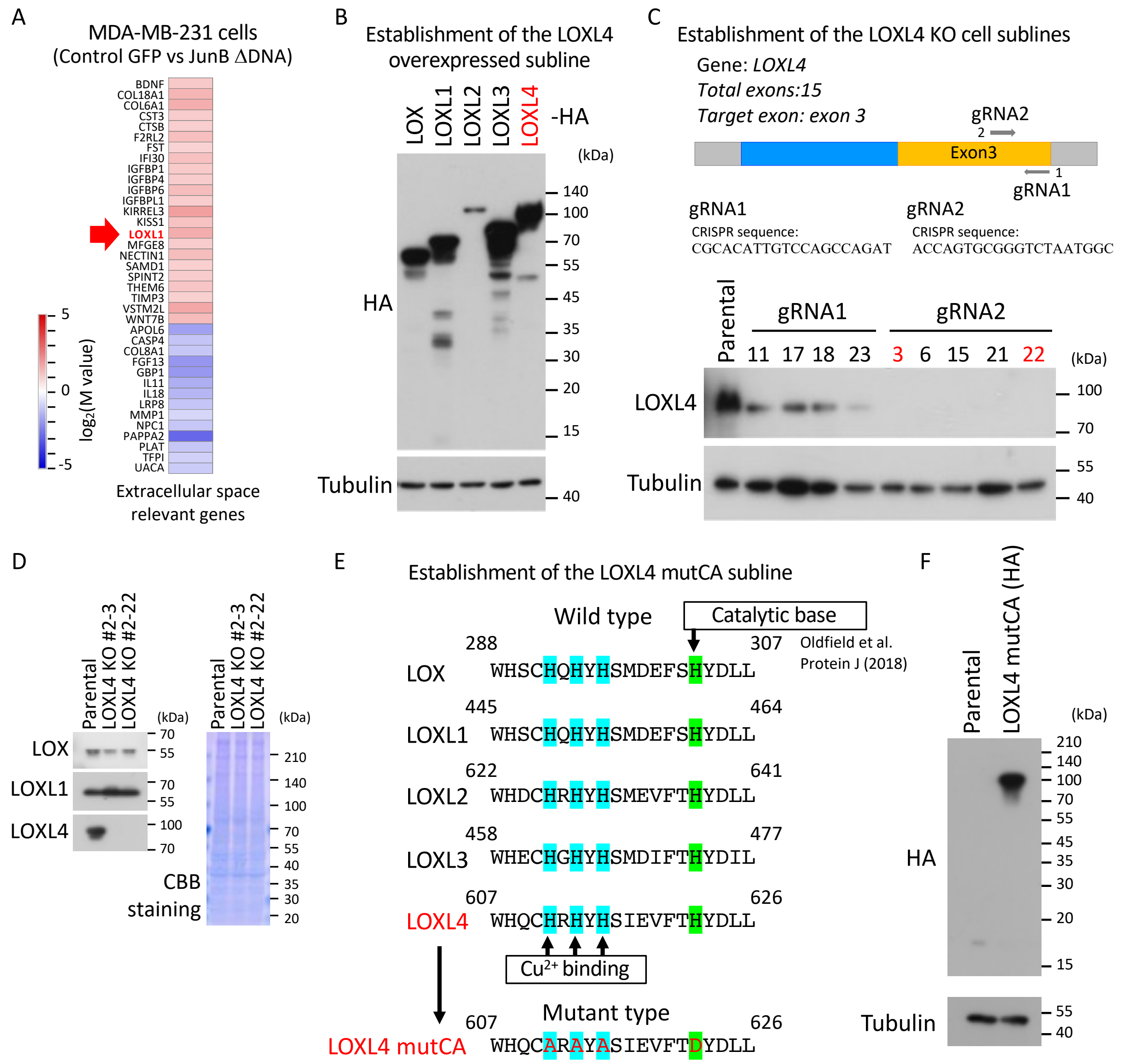

Suppl. Fig. S1. Establishment of the genetically engineered sublines based on MDA-MB-231 cells as parental cells. A: An RNAseq analysis was performed for the stable subline of MDA-MB-231 cells that overexpress JunB ΔDNA in a comparison with the control green fluorescence protein (GFP)-overexpressing subline. The heat map shows the selected

extracellular space-relevant genes that are upregulated or downregulated. **B:** Establishment of MDA-MB-231-derived sublines that stably express foreign LOX, LOXL1, LOXL2, LOXL3, or LOXL4, which were all designed to have an HA tag at the C-terminal side. **C:** Establishment of LOXL4 knock-out (KO) cells according to the CRISPR/Cas9 method. The target sequences for the genetic ablation of *LOXL4* gene were designed and applied as guide (g)RNA1 and gRNA2. To obtain the optimal clones that show the complete deletion of the expression of LOXL4, we determined the expression levels of the LOXL4 at the protein level, and we observed that gRNA2 was very effective genetically erasing the expression of intrinsic *LOXL4* gene. We thus picked up two clones (#2-3 and #2-22) from the gRNA2-clones' pool. **D:** To confirm the unexpected raising of any off-target silencing in the LOXL4 KO cells, another LOX family molecule LOXL1, which we have recently reported to express in MDA-MB-231 cells at high levels and to play a crucial role in the upregulation of invasiveness of MDA-MB-231 cells (Hirabayashi et al. *Front Oncol* 2023), was analyzed for their expressions in the KO cells by the WB procedure. The expression levels of LOXL1 were not altered in the two KO cell sublines compared to their parental MDA-MB-231 cells. No altered expression was observed for the additional LOX family protein LOX among the indicated cells. **E:** On the basis of the well conserved catalytic motif among the LOX family members, the essential amino acid of the catalytic activity in LOXL4, i.e., 622-histidine (622H) (*green*) and the essential amino acids to capture Cu^{2+} in the motif, i.e., 611H, 613H, and 615H (*blue*) were all replaced with the indicated amino acids, resulting in the LOXL4 mutant construct named LOXL4 mutCA. **F:** After the force-delivery of the construct into MDA-MB-231 cells, a stable subline that overexpressed LOXL4 mutCA was established. The expression of the foreign LOXL4 mutCA was confirmed by WB.

Hirabayashi D, Yamamoto K-I, Maruyama A, Tomonobu N, Kinoshita R, Chen Y, et al. LOXL1 and LOXL4 are Novel Target Genes of the Zn^{2+}-Bound Form of ZEB1 and Play as Crucial Role in the Acceleration of Invasive Events in Triple-Negative Breast Cancer Cells. *Front Oncol* (2023) 13:1142886. doi:10.3389/fonc.2023.1142886.

Suppl. Fig. S2

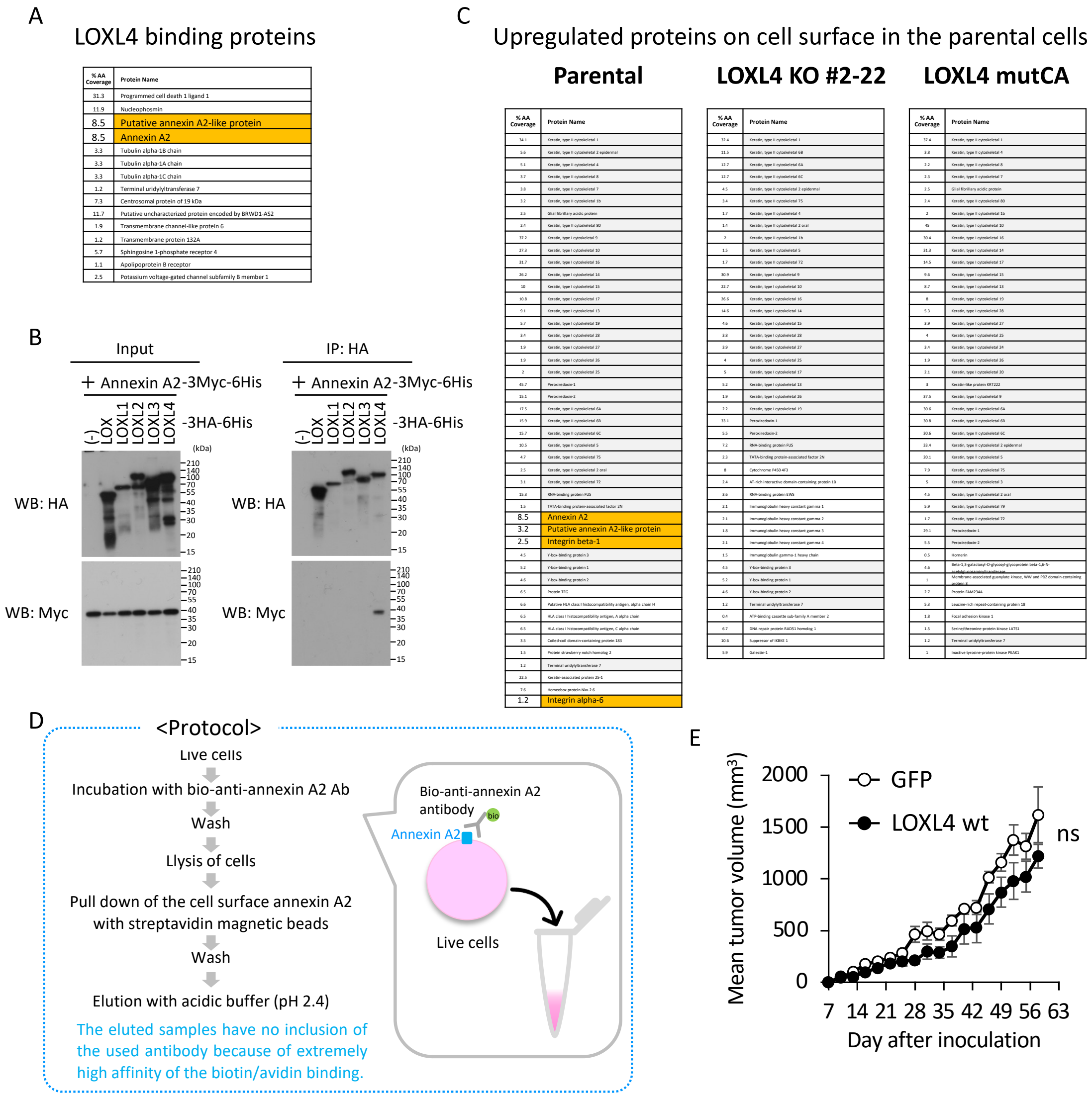

Suppl. Fig. S2. Identification of the LOXL4 regulatory molecule(s). A: To explore novel binding partners of LOXL4 on the cell surface, we transfected HEK293T cells with the plasmid carrying the HA-tagged LOXL4 wt. The conditioned medium from the transfected cells was collected and added to the MDA-MB-231 culture and maintained for 24 hr. The incubated MDA-MB-231 cells were then collected, lysed, and subjected to the

immunoprecipitation procedure using anti-HA-tag antibody-conjugated agarose beads. The collected immunoprecipitated was eluted from the beads by an acidic buffer (100 mM glycin-HCl buffer, pH 2.0), neutralized with 1.0 M Tris-HCl buffer (pH 9.0), dialyzed with trypsin digestion buffer (10 mM $CaCl_2$, 100 mM ammonium bicarbonate; pH 7.8), and trypsinized overnight at 37°C. The digested proteins were directly subjected to a shotgun-type protein identification approach using a nano-flow liquid chromatography-mass spectrometry apparatus (Agilent 6330 Ion Trap, Agilent Technologies, Santa Clara, CA) equipped with an analytical chip (Agilent HPLC-Chip, Agilent Technologies). The resulting tandem mass spectrometry spectra of the tryptic peptides were finally analyzed using the Agilent software program Spectrum Mill MS Proteomics Workbench with the protein database (SwissPlot) for putative Homo sapiens protein identifications. The identified proteins in the procedure using the conditioned medium of the control GFP transfectant were deleted from the LOXL4 binding list. **B:** To study the specificity of the binding of LOXL4 to annexin A2, we individually combined the plasmids carrying each LOX family member tagged with HA-tag with the Myc-tagged annexin A2 plasmid, and they were then transfected to HEK293T cells. The transfected cells were lysed and used to conduct immunoprecipitations with anti-HA tag antibody-conjugated agarose beads. After the immunoprecipitation of the expressed products with the beads, the bound foreign annexin A2 was western-blotted by the anti-Myc antibody. **C:** To identify the cell surface proteins comprehensively, the indicated live cells were all treated with sulfosuccinimidyl 3-[[2-(biotinamido) ethyl] dithio] propionate (Toronto Research Chemicals, Toronto, ON, Canada) to label membrane proteins under the living cell conditions. The treated cells were collected, lysed, and immunoprecipitated with anti-biotin antibody-conjugated agarose beads (Sigma Aldrich). The subsequent elution and tandem

mass spectrometry (MS/MS) procedure were followed by a method similar to that described in panel A. This protocol was used to obtain the data presented **Fig. 4C** and with some modifications to the data in **Fig. 4D,E**. The *gray-colored columns* are all overlapped proteins identified together. **D:** The collection of the cell surface annexin A2 in accord with our designed protocol. **E:** Tumor growth was assessed in an orthotopic xenograft model. A tumor growth curve was established after an injection of the indicated cells (control GFP and LOXL4 wt sublines), and the tumor diameters were measured. Data are mean ± SEM (n=4). ns: no significance.

frontiers
